# Efficacy of antimicrobial prophylaxis on the risk of surgical site infections in companion animal surgery: a systematic review and meta‐analysis for European Network for Optimization of Antimicrobial Therapy (ENOVAT) guidelines

**DOI:** 10.1111/jsap.70055

**Published:** 2026-02-18

**Authors:** T. M. Sørensen, K. Scahill, J. S. Weese, F. Allerton, L. R. Jessen, from the European Network for Optimization of Antimicrobial Therapy (ENOVAT) guidelines and the ESCMID Study Group for Veterinary Microbiology (ESGVM)

**Affiliations:** ^1^ Department of Veterinary Clinical Sciences University of Copenhagen Frederiksberg C Denmark; ^2^ ESCMID Study Group for Veterinary Microbiology (ESGVM) Basel Switzerland; ^3^ College of Medicine and Veterinary Medicine University of Edinburgh Edinburgh UK; ^4^ Evidensia Södra Djursjukhuset Kungens Kurva Kungens Kurva Sweden; ^5^ Department of Pathobiology, Ontario Veterinary College University of Guelph Guelph Ontario Canada; ^6^ Willows Veterinary Centre & Referral Service Shirley UK

## Abstract

**Objectives:**

Like all antimicrobial use, surgical antimicrobial prophylaxis should only be administered when the benefits outweigh potential harms. The aim of this systematic review and meta‐analysis was to assess the effectiveness of surgical antimicrobial prophylaxis to reduce surgical site infections in dogs and cats.

**Materials and Methods:**

Population, intervention, comparator and outcome were generated by a multidisciplinary guideline panel and clinical decision thresholds were derived from stakeholder interviews. The Grading of Recommendations Assessment, Development and Evaluation methodology was used to evaluate the certainty of the evidence.

**Results:**

Eight randomised controlled trials and seven observational studies met the eligibility criteria and were included in the review. Nine categories of surgical procedures were created based on organ system and wound classification and outcomes included surgical site infections, adverse events and mortality. Very low to moderate certainty evidence showed that surgical antimicrobial prophylaxis only had a trivial or small clinical effect on surgical site infection incidence in all surgical procedures. No adverse effects or mortalities related to surgical site infections were reported in any of the studies.

**Clinical Significance:**

The results of this study will be used to create evidence‐based treatment guidelines.

## INTRODUCTION

Surgical antimicrobial prophylaxis (SAP) is administered to prevent or minimise the risk of surgical site infections (SSIs) (Billings et al., 1990; Cockburn et al., 2022; Launcelott et al., 2019; Vasseur et al., 1988). With high hygiene standards in the peri‐operative handling of patients, SSIs represent an infrequent but still potentially serious complication of any surgical intervention with negative consequences for the affected animal’s health (including increased mortality), pet owner satisfaction and clinician confidence. Any measure that can reduce the likelihood of a SSI will likely be appreciated by the surgeon, but the severity and consequences of any SSI also depend on the site affected. The majority of SSI are superficial, may be managed with topical treatment and are without serious impact on patient morbidity or mortality. The prophylactic administration of antimicrobials has therefore become controversial because of negative effects including adverse drug reactions, promotion of antimicrobial resistance and disruption of the bacterial microbiota (Menz et al., 2021; Stavroulaki et al., 2023). To justify SAP, antimicrobial administration should significantly reduce morbidity and/or mortality tipping the balance towards demonstrable benefits.

Current national guidelines and recommendations for SAP for most procedures are based on expert opinion and do not reflect a comprehensive analysis of the available evidence (Allerton et al., 2021). Recently, a scoping review mapped the current body of evidence for SAP in veterinary practice (Sørensen et al., 2024) showing that available evidence is dominated by observational studies, predominantly reporting orthopaedic procedures with relatively few randomised controlled trials.

The primary aim of this systematic review and meta‐analysis was to evaluate the efficacy of SAP on the risk of SSI after companion animal surgical procedures. The results will inform the SAP Antimicrobial Use Guidelines of the European Network for Optimization of Antimicrobial Therapy (ENOVAT) and European Society of Clinical Microbiology and Infectious Diseases (ESCMID) Study Group for Veterinary Microbiology (ESGVM). The ENOVAT guidelines initiative encourages the development of regional or national antimicrobial treatment guidelines for companion animal SAP based on the results of this study.

## MATERIALS AND METHODS

This systematic review and meta‐analysis is based on a recently published scoping review (Sørensen et al., 2024) and is reported in adherence with the Preferred Reporting Items for Systematic Review and Meta‐Analysis (PRISMA) statement (Page et al., 2021). No separate protocol was registered for this systematic review.

### Population, intervention, comparator, outcome (PICO) question generation and importance of outcomes

The surgical procedures included in the systematic review were selected by the ENOVAT Surgical prophylaxis guideline panel. Procedures were prioritised by the panel in an iterative process using an electronic Delphi questionnaire until agreement was reached (>80% strongly agreeing or agreeing on their inclusion). PICO questions were drafted by thematically grouping surgical procedures according to organ system and wound classification. SSI development was selected as the primary critical outcome measure with 100% agreement from the guideline panel.

### PICOs and subgroups

Parallel PICO questions were phrased to allow separate consideration of the need for peri‐operative SAP and post‐operative SAP reflecting the different decision points.

PICO 1: In dogs and cats undergoing a specified surgical procedure, does peri‐operative antimicrobial prophylactic administration compared to no antimicrobial administration reduce the risk of post‐operative SSI?

PICO 2: In dogs and cats undergoing a specified surgical procedure, does post‐operative antimicrobial prophylactic administration compared to no antimicrobial administration reduce the risk of post‐operative SSI?

The population of dogs and cats undergoing surgery was categorised into nine subgroups (S1 to S9) according to surgical procedure and wound classification (Table 1). Selected outcomes were prioritised as proportion of SSI, all‐cause mortality and adverse events.

**Table 1:** Subgroup categorisation of surgical procedures (S1 to S9) by surgery group and wound classifications

Surgical sites	Wound classification	Subgroup	
Soft tissue surgery	Clean wounds No signs of inflammation No involvement of respiratory, GI or urogenital* tracts	S1	Neutering
		S2	Other clean soft tissue
	Clean‐contaminated Low level of contamination Controlled entry into respiratory, alimentary or urogenital tract	S3	Urologic
		S4	GI
		S5	Other clean‐contaminated soft tissue
	Contaminated Breach in sterile techniques Leakage from GI tract	S6	Contaminated
Orthopaedic surgery	Clean or clean‐contaminated	S7	Orthopaedic without implants
		S8	Orthopaedic with implants
		S9	TPLO

GI, Gastrointestinal; TPLO, Tibial plateau levelling osteotomy

* Apart from neutering.

### Definitions

Applied SSI definitions were adjusted based on the Center for Disease Control and Preventions (CDC) National Healthcare Safety Network (NHSN) classifications shown in Table 2. In the categorisation of studies based on SAP timing, peri‐operative administration was defined as administration of antimicrobials from 2 hours before the surgical incision until 24 hours after surgical wound closure. Post‐operative administration was defined as antimicrobial administration beyond 24 hours after surgical wound closure.

**Table 2:** Applied definitions of surgical site infections based on timing, location and extent of the infection

Type of SSI	Definition	Must meet at least one of the following criteria
Superficial incisional	Occurs within 30 days after the surgical procedure Involve only skin and SC tissue	Purulent discharge from the superficial incision Organism(s) identified from aseptically obtained specimen from superficial incision or SC tissue Superficial incision is deliberately opened by the surgeon AND at least one of the following signs: Localised pain, swelling, erythema or heat Diagnosis of a superficial incisional SSI by the surgeon
Deep incisional	Occurs within 30 days after the surgical procedure, or 1 year if an implant is left in place Involves deep soft tissue (e.g. fascia, muscle)	Purulent discharge from the deep incision Organism(s) identified from aseptically obtained specimen from deep soft tissues AND incision spontaneous dehisces or is deliberately opened by the surgeon AND at least one of the following signs: Localised pain, swelling, erythema or heat An abscess or other infection involving the deep incision is detected on gross examination, histopathology or imaging
Organ/Space	Occurs within 30 days after the surgical procedure, or 1 year if an implant is left in place Involves any part of the body deeper than the fascial or muscle layers that is opened during the surgery	Purulent discharge from a drain that is placed through a stab wound into the organ or space Organism(s) identified from aseptically obtained specimen from organ or space An abscess or other infection involving the organ or space is detected on gross examination, histopathology or imaging

Adjusted from the Center for Disease Control and Prevention classification of surgical site infections. Based on Center for Disease Control and Preventions National Healthcare Safety Network wound classification. Table reproduced from Low et al. (2022).

SC Subcutaneous; SSI Surgical site infection

### Clinical thresholds

The relevance of the estimated effects of antimicrobial use on SSI rates, as derived from the meta‐analysis, was evaluated by comparison with clinical decision thresholds derived from interviews with target stakeholders (Hultcrantz et al., 2017). Eight guideline panel members and 16 veterinary practitioners from across Europe who frequently perform surgical procedures were interviewed and completed a structured series of questions. Participants were presented with estimated baseline risks of superficial, deep, organ/space or implant‐associated SSIs (as defined by consensus opinion of the panel) for specified surgical procedures and a potential risk reduction that could be obtained with SAP.

Respondents were first asked if they would treat 1000 dogs/cats, undergoing the example procedure, with SAP (peri‐ or post‐operative) to reduce the (superficial) SSI rate from 50 (5%) to 5 (0.05%). If they accepted the proposed risk reduction, they were then asked to define the smallest risk reduction (number per 1000 treated animals) that they would continue to use SAP (i.e. they would treat 1000 dogs/cats with SAP to avoid “X” SSIs). Respondents who rejected the proposed risk reduction were asked to define the smallest risk reduction (number per 1000 treated animals) where they would use SAP (i.e. they would treat 1000 dogs/cats with SAP to avoid “X” SSIs). Small, moderate and large effect thresholds were established by calculating the 25th percentile of the responses to questions around organ/space/implant, deep and superficial SSI, respectively. The moderate thresholds were calculated as the value in between the small and large effect threshold if no difference was indicated by respondents (S1) or where deep SSI was not included in the survey (S7 to S9). The threshold interview guide and questionnaire can be found in Supplementary file 1. Clinical thresholds are summarised in Table 3.

**Table 3:** Clinical decision thresholds applied during the guideline recommendation process

Subgroups	SAP	Thresholds			Baseline SSI acceptance	
		Small effect	Moderate effect	Large effect	Superficial SSI	Deep/implant/space SSI
S1	Peri‐OP	50	125	200	97%	97%
	Post‐OP	50	100	250	100%	100%
S2 and S3	Peri‐OP	23	50	200	97%	84%
	Post‐OP	30	100	250	100%	97%
S4	Peri‐OP	30	40	200	97%	27%
	Post‐OP	30	70	250	100%	54%
S5	Peri‐OP	30	50	200	97%	19% to 54%
	Post‐OP	30	100	250	100%	59% to 73%
S7 to S9	Peri‐OP	20	60	100	68%	64% to 78%
S7	Post‐OP	50	125	200	92%	78%
S8 and S9	Post‐OP	20	110	200	92%	64%

Threshold numbers are the number of animals with a change in outcome (SSI absolute effect size), due to an intervention (surgical antimicrobial prophylaxis) in 1000 procedures/animals, that veterinarians deemed clinically relevant. Acceptance of baseline SSI levels without the use of antimicrobials is shown as the proportion of acceptance among survey respondents

AM Antimicrobials; OP Operative; SAP Surgical antimicrobial prophylaxis; SSI Surgical site infection; S1 Subgroup 1 (neutering); S2 Subgroup 2 (other clean soft tissue); S3 Subgroup 3 (Urologic); S4 Subgroup 4 (gastrointestinal); S5 Subgroup 5 (other clean‐contaminated soft tissue); S7 Subgroup 7 (orthopaedic without implants); S8 Subgroup 8 (orthopaedic with implants); S9 Subgroup 9 (Tibial plateau levelling osteotomy)

### Search strategy, eligibility criteria, study selection and data collection

The search strategy and original eligibility criteria is fully described in the scoping review (Sørensen et al., 2024). In brief, Cambridge Agricultural and Biological Abstracts (CAB Abstracts), EMBASE, MEDLINE, Scopus and Web of Science Core Collection were searched. At least two reviewers independently screened titles and abstracts or evaluated full texts in an online tool for systematic reviews (Ouzzani et al., 2016). Predetermined data (country of origin, language, investigation year, design, setting and follow‐up period, population, group size, species, surgical procedures, diagnoses, intervention substance, dosage, frequency and timing, assessment methodology, SSI rate, mortality and adverse effect) were extracted independently into a data management tool (Excel, Microsoft). Observational studies were included as a complement due to the low and very low level of certainty of the randomised trials (Cuello‐Garcia et al., 2022). Papers were eligible to be included in the systematic review and meta‐analysis if they:

- were prospective studies (RCT or observational)
- involved soft tissue or orthopaedic surgical procedures performed on companion animals (dogs and/or cats) and
- described timing of SAP and post‐operative SSI for treated and untreated animals separately, or this information was available from the corresponding author.

### Data synthesis

To estimate the pooled effect size across studies, a direct pairwise meta‐analysis was performed. RevMan 5.4 (The Nordic Cochrane Center, The Cochrane Collaboration) was used for meta‐analysis and generation of forest plots to quantify and illustrate the differences between the intervention and comparator groups and assess their significance. SSI as a dichotomous outcome were entered to estimate the risk ratio (RR) from inverse variance fixed effect statistical model to give larger studies with smaller variances more weight than smaller studies with larger variances. Heterogeneity among included studies was evaluated using the *I* ^2^ statistic and by visual inspection to identify outliers (Guyatt et al., 2023). Forest plots were generated to visually show the results of individual studies and subgroups alongside the overall pooled estimate. Summary of finding (SoF) tables including anticipated absolute effects for dichotomous outcomes were generated through the GRADEpro online software (GRADE, 2023) after importation of the RevMan files.

### Quality assessment

The quality of the evidence was assessed with the Grading of Recommendations Assessment, Development and Evaluation (GRADE) methodology (Guyatt et al., 2008), hereafter referred to as the “certainty of evidence” in accordance with GRADE terminology. The certainty of evidence assessment was based on randomised studies. Observational studies were included for complimentary purposes and were reported separately. The certainty of evidence interpretation and description of GRADE domains included in the evaluation of the evidence is presented in Table 4. The domains were assessed for all individual subgroups (S1 to S9). A contextualised approach was used for imprecision and inconsistency domain assessments (Guyatt et al., 2023; Schünemann et al., 2022). Imprecision was assessed based on absolute effects calculated in the SoF tables. The risk difference (RD) was calculated by applying the relative effects to a measure of baseline risk. The effect estimate was considered precise if the 95% CI did not cross any of the predefined thresholds and considered imprecise if the 95% CI crossed one or more thresholds, as decision‐making could be affected should the true effect lie outside the 95% CI. The number of threshold limits crossed by the 95% CI determined the severity of the imprecision.

**Table 4:** Definitions for Certainty of evidence and short descriptions of the five domains used to grade the evidence using the Grading of Recommendations Assessment, Development and Evaluation (GRADE) approach

Definition	Description
Certainty*	
Very low	The true effect is probably markedly different from the estimated effect
Low	The true effect might be markedly different from the estimated effect
Moderate	The authors believe that the true effect is probably close to the estimated effect
High	The authors have a lot of confidence that the true effect is similar to the estimated effect
Domains^†^	
Risk of bias	Bias occurs when the results of a study do not represent the truth because of inherent limitations in design or conduct of a study. The body of evidence is rated at the outcome level rather than the study level
Inconsistency	When several studies show consistent effects the certainty of evidence is higher. Similarity of point estimates and the overlap of CI are evaluated in the rating process
Indirectness	Evidence is most certain when studies directly compare the interventions of interest in the population of interest, and report the outcome(s) critical for decision‐making. Certainty can be rated down if the individuals studied are different from those that the recommendation refers to. Indirectness can also occur when the interventions studied are different than the real outcomes
Imprecision^‡^	This domain was recently updated and relies on thresholds and CI of absolute effects as a primary criterion for imprecision rating (i.e., CI approach). Certainty is downgraded when confidence intervals cross predefined clinical thresholds (trivial, small, moderate and large) that are of importance to stakeholders. If the relative effect is large the optimal information size is also considered
Other considerations	Publication bias can contribute to further downgrading, but the consideration of large effects, plausible confounding and dose–response gradients can occasionally lead to upgrading of the certainty of evidence

Table reproduced from Scahill et al. (2024)

CI, confidence intervals

* Cited from Guyatt et al. (2008)

^†^ Adapted from Guyatt et al. (2008)

^‡^ Adapted from Schünemann et al. (2022)

Two assessors applied the Cochrane risk‐of‐bias tool for randomised trials (RoB 2.0) to assess the risk of bias in each of the RCT studies for the SSI outcome. There was a critical risk of uncontrolled confounding in observational studies and a decision was made to not proceed with the risk of bias tool for non‐randomised studies (ROBINS‐I V2). All observational studies were therefore considered to be of high risk of bias.

## RESULTS

### PICO 1

*In dogs and cats undergoing surgery, does peri‐operative antimicrobial prophylactic administration compared to no antimicrobial administration reduce the risk of post‐operative SSI?*

#### Included studies

The scoping review (Sørensen et al., 2024) identified eight relevant prospective studies including four RCTs and four comparative observational studies (Table 5). Administration of peri‐operative antimicrobial prophylaxis represented the intervention in all included RCTs but none of the observational studies. The eight studies included a total of 3480 privately owned dogs and cats and there were no baseline differences regarding age, breed and sex between intervention and comparator groups across the studies.

**Table 5:** Study characteristics for studies reporting incidence of surgical site infections in dogs and cats administered peri‐operative antimicrobial prophylaxis versus dogs and cats not administered antimicrobial prophylaxis peri‐operatively (PICO 1)

Study	Subpopulation	Intervention			Comparator		Clinical outcome assessment	
		*n*	Substance	Dose, route	*n*	Placebo	Follow‐up	SSI definition
Daude‐Lagrave et al. (2001)*	S1, S2, S3, S4, S5, S7, S8	446	Cefalexin	30 mg/kg iv at induction and 4 hours later	427	NaCl iv	12 days	Wound drainage and/or body temp >39.5, wound characteristics (redness, heat, swollen, local induration, possible oozing)
Holmberg (1985)*	S8	30	Penicillin procaine	40,000 U/kg im and iv at induction, at closure 50,000 U/kg Topical	30	None	6 months	NR
Vasseur et al. (1985)*	S1, S2, S7, S8	64	Ampicillin	20 mg/kg at induction and 4 hours later	64	NaCl iv	7 to 10 days	Wound drainage
Whittem et al. (1999)*	S8	91	Cefazolin Penicillin G	20 mg/kg iv or 40,000 U/kg iv 30 minutes pre‐surgery and q90m	35	Placebo NaCl iv	10 to 14 days	NR
Brown et al. (1997)	S1, S2, S6	169	NR	NR	777	NR	14 days	Purulent discharge
Castro et al. (2022)	S1	10	Cefalothin	30 mg/kg im pre‐OP	10	NR	7 days	NR
Stetter et al. (2021)	S1	79	NR	NR	432	NR	30 days	Purulent discharge/positive culture/one or more inflammation signs AND reoperation
Turk et al. (2015)	S1 to S6	802	NR	NR	44	NR	30 days/1 year^†^	Purulent discharge/positive culture/one or more inflammation signs AND reoperation

AM Antimicrobial; AMC Amoxicillin+clavulanic acid or potentiated amoxicillin; bid Twice daily; CDC Center for Disease Control and Prevention; iv Intravenous; NR Not reported; NA Not applicable; NaCl Sodium chloride; NR Not reported; OP Operative; po Per oral; sc Subcutaneous; SSI Surgical site infection; S1 Subgroup 1 (neutering); S2 Subgroup 2 (other clean soft tissue); S3 Subgroup 3 (urologic); S4 Subgroup 4 (gastrointestinal); S5 Subgroup 5 (other clean‐contaminated soft tissue); S6 Subgroup 6 (contaminated); S7 Subgroup 7 (orthopaedic without implants); S8 Subgroup 8 (orthopaedic with implants); S9 Subgroup 9 (tibial plateau levelling osteotomy)

* Randomised controlled trials

^†^ 1 year for implant‐related SSI

In four studies, data were only classified to the level of surgical wound categories; individual surgical procedure subgroups could not be identified (Brown et al., 1997; Daude‐Lagrave et al., 2001; Turk et al., 2015; Vasseur et al., 1985), data were no longer available (Turk et al., 2015) or it was not possible to reach the authors. No studies were found where data could be extracted for dogs representing the S9 subgroup specifically. Individual study characteristics are presented in Table 5.

#### Effect of antimicrobial prophylaxis versus no antimicrobial prophylaxis

**FIG 1:**
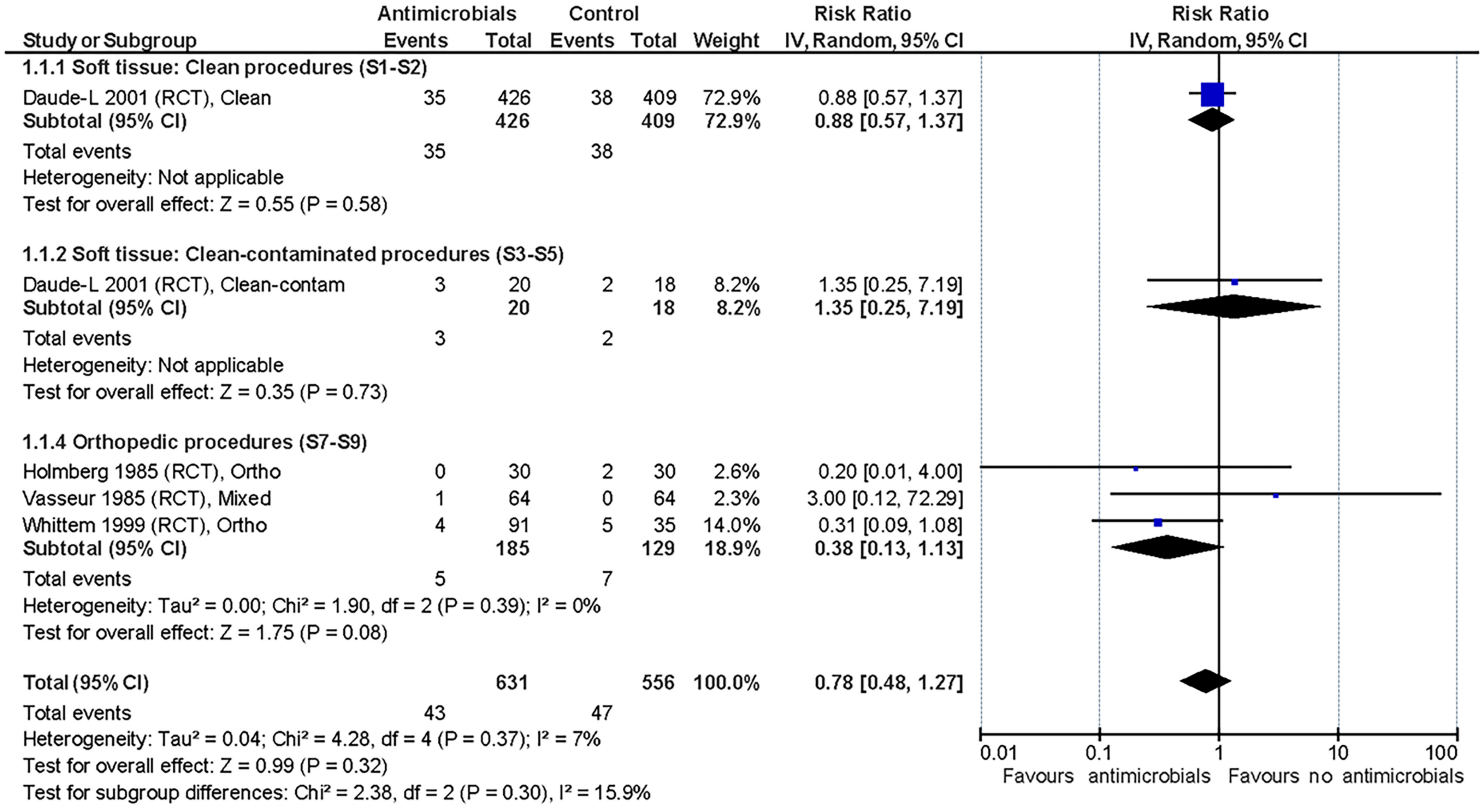
Randomised controlled trials included for evaluation of effect of peri‐operative surgical antimicrobial prophylaxis (SAP) on surgical site infections (SSIs). Forest plot of the relative risk for SSI in dogs and cats administered peri‐operative SAP versus dogs and cats not administered antimicrobial prophylaxis. Trials are subgrouped (S1 to S9) by surgery and wound classifications (Table 1). Individual trial results are shown as blue squares and pooled effects are shown as black diamonds. Relative risks are converted to absolute risks and related to clinical thresholds in Table 6. S1, Subgroup 1 (neutering); S2, Subgroup 2 (other clean soft tissue); S3, Subgroup 3 (urologic); S4, Subgroup 4 (gastrointestinal); S5, Subgroup 5 (other clean‐contaminated soft tissue); S7, Subgroup 7 (orthopaedic without implants); S8, Subgroup 8 (orthopaedic with implants); and S9, Subgroup 9 (tibial plateau levelling osteotomy).

**FIG 2:**
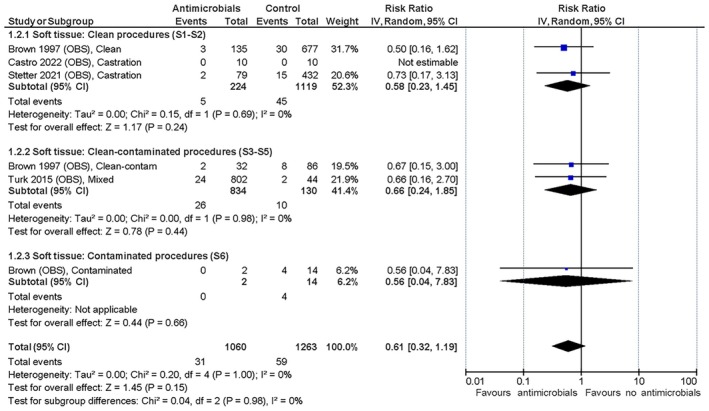
Observational studies on soft tissue surgical procedures included for evaluation of effect of peri‐operative surgical antimicrobial prophylaxis (SAP) on surgical site infections (SSIs). Forest plot of the relative risk for SSI in dogs and cats administered peri‐operative SAP versus dogs and cats not administered antimicrobial prophylaxis. Studies are subgrouped (S1 to S9) by surgery and wound classifications (Table 1). Individual study results are shown as blue squares and pooled effects are shown as black diamonds. Relative risks are converted to absolute risks and related to clinical thresholds in Table 6. S1, Subgroup 1 (neutering); S2, Subgroup 2 (other clean soft tissue); S3, Subgroup 3 (urologic); S4, Subgroup 4 (gastrointestinal); S5, Subgroup 5 (other clean‐contaminated soft tissue); and S6, Subgroup 6 (contaminated).

**FIG 3:**
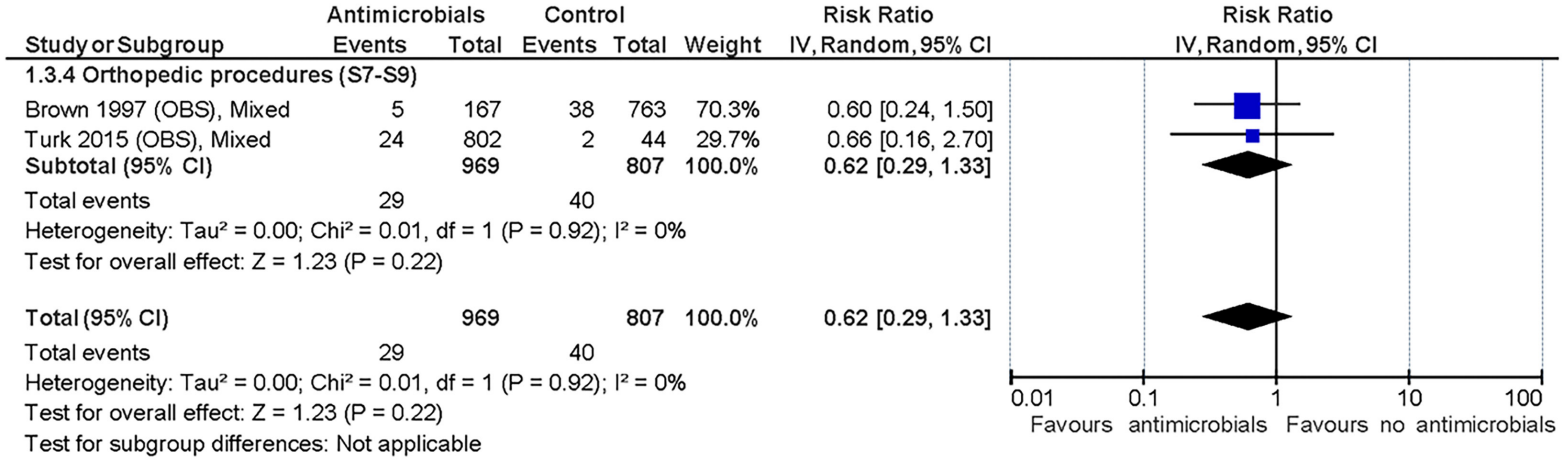
Observational studies on orthopaedic surgical procedures included for evaluation of effect of peri‐operative surgical antimicrobial prophylaxis (SAP) on surgical site infections (SSIs). Forest plot of the relative risk for SSI in dogs and cats administered peri‐operative SAP versus dogs and cats not administered antimicrobial prophylaxis. Studies are subgrouped (S7 to S9) by surgery and wound classifications (Table 1). Individual study results are shown as blue squares and pooled effects are shown as black diamonds. Relative risks are converted to absolute risks and related to clinical thresholds in Table 6. S7, Subgroup 7 (orthopaedic without implants); S8, Subgroup 8 (orthopaedic with implants); and S9, Subgroup 9 (tibial plateau levelling osteotomy).

The only RCT with primarily clean, soft tissue procedures found a trivial effect of the peri‐operative administration of SAP (Daude‐Lagrave et al., 2001) on SSI development, as did the three non‐RCT studies (Brown et al., 1997; Castro et al., 2022; Stetter et al., 2021). The three RCT and two non‐RCT studies with solely or primarily orthopaedic procedures showed a trivial to small effect of the intervention in favour of peri‐operative SAP (Brown et al., 1997; Holmberg, 1985; Turk et al., 2015; Vasseur et al., 1985; Whittem et al., 1999) with relative risk reductions (RRRs) of 38% to 63% and a number needed to treat (NNT) between 29 and 53 (you have to treat 29 to 53 animals prophylactically to avoid one additional SSI) based on the included evidence. The summarised relative effects are shown in Figs 1, 2, 3 and absolute effects in Table 6. SoF tables can be found in Supplementary file 2). Mortality was reported in all studies, but none were deemed associated with SSI development. No studies reported adverse effects attributed to antimicrobial use.

**Table 6:** The relative and absolute effects of peri‐operative surgical antimicrobial prophylaxis versus no surgical antimicrobial prophylaxis

PICO 1	Study design	Risk ratio (95% CI)	*P*‐value	Absolute effect of peri‐operative treatment	Clinical relevance*	Certainty of evidence
S1	RCT	0.88 [0.57 to 1.37]	.58	11 fewer SSI per 1000 animals (95% CI from 40 fewer to 34 more)	Trivial effect	Low
	OBS	0.58 [0.23 to 1.45]	.24	17 fewer SSI per 1000 animals (95% CI from 31 fewer to 18 more)	Trivial effect	Very low
S2	RCT	0.88 [0.57 to 1.37]	.58	11 fewer SSI per 1000 animals (95% CI from 40 fewer to 34 more)	Trivial effect	Very low
	OBS	0.58 [0.23 to 1.45]	.24	17 fewer SSI per 1000 animals (95% CI from 31 fewer to 18 more)	Trivial effect	Very low
S3	RCT	1.35 [0.25 to 7.19]	.73	39 more SSI per 1000 animals (95% CI from 83 fewer to 688 more)	Small effect	Very low
	OBS	0.66 [0.24 to 1.85]	.44	26 fewer SSI per 1000 animals (95% CI from 58 fewer to 65 more)	Small effect	Very low
S4	RCT	1.35 [0.25 to 7.19]	.73	39 more SSI per 1000 animals (95% CI from 83 fewer to 688 more)	Small effect	Very low
	OBS	0.66 [0.24 to 1.85]	.44	26 fewer SSI per 1000 animals (95% CI from 58 fewer to 65 more)	Trivial effect	Very low
S5	RCT	1.35 [0.25 to 7.19]	.73	39 more SSI per 1000 animals (95% CI from 83 fewer to 688 more)	Small effect	Very low
	OBS	0.66 [0.24 to 1.85]	.44	26 fewer SSI per 1000 animals (95% CI from 58 fewer to 65 more)	Trivial effect	Very low
S6	OBS	0.56 [0.04 to 7.83]	.66	126 fewer SSI per 1000 animals (95% CI from 274 fewer to 1951 more)	NA	Very low
S7	RCT	0.38 [0.13 to 1.13]	.22	34 fewer SSI per 1000 animals (95% CI from 47 fewer to 7 more)	Small effect	Very low
	OBS	0.62 [0.29 to 1.33]	.08	19 fewer SSI per 1000 animals (95% CI from 35 fewer to 5 more)	Trivial effect	Very low
S8	RCT	0.38 [0.13 to 1.13]	.22	34 fewer SSI per 1000 animals (95% CI from 47 fewer to 7 more)	Small effect	Very low
	OBS	0.62 [0.29 to 1.33]	.08	19 fewer SSI per 1000 animals (95% CI from 35 fewer to 5 more)	Trivial effect	Very low
S9	RCT	0.38 [0.13 to 1.13]	.22	34 fewer SSI per 1000 animals (95% CI from 47 fewer to 7 more)	Small effect	Very low
	OBS	0.62 [0.29 to 1.33]	.08	19 fewer SSI per 1000 animals (95% CI from 35 fewer to 5 more)	Trivial effect	Very low

The clinical relevance is interpreted according to predetermined thresholds (Table 3) and the certainty of evidence according to the GRADE approach (Table 4)

CI Confidence interval; GRADE Grading of Recommendations Assessment, Development and Evaluation; NA Not applicable; OBS Observational trial; RCT Randomised controlled trial; S1, Subgroup 1 (neutering); S2, Subgroup 2 (other clean soft tissue); S3, Subgroup 3 (urologic); S4, Subgroup 4 (gastrointestinal); S5, Subgroup 5 (other clean‐contaminated soft tissue); S7, Subgroup 7 (orthopaedic without implants); S8, Subgroup 8 (orthopaedic with implants); and S9, Subgroup 9 (tibial plateau levelling osteotomy).

* The clinical relevance of the estimated absolute effect is interpreted according to the thresholds selected by the veterinarians/stakeholders

#### Certainty of evidence

**FIG 4:**
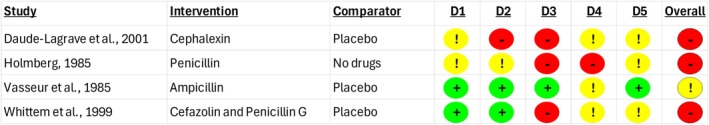
Risk of bias analysis for randomised controlled trials included in the review on peri‐operative administration of antimicrobials for surgical site infection prophylaxis. D1, Randomisation process; D2, Deviations from the intended interventions; D3, Missing outcome data; D4, Measurement of the outcome; and D5, Selection of the reported result. Green dots indicate low risk of bias, yellow dots some concerns and red dots high risk of bias.

For the four RCT studies comparing peri‐operative SAP, there were concerns of high risk of bias, primarily because of a lack of reporting relevant details on methodology as shown in Fig 4.

Consequently, the risk of bias was downgraded two levels in the certainty assessment. All included studies were deemed to have very serious risk of bias. The overall certainty of the evidence was very low in all subgroups except neutering (S1) primarily due to the very serious risk of bias. However, serious or extremely serious imprecision was found because of the wide 95% CI for most effect estimates, and serious indirectness was found for some subgroups as not all surgical subgroups were represented or could be satisfyingly extracted from the published data. Full certainty of evidence decisions can be found in Supplementary file 3.

### PICO 2

*In dogs and cats undergoing surgery, does post‐operative antimicrobial prophylactic administration compared to no antimicrobial administration reduce the risk of post‐operative SSI?*

#### Included studies

Seven prospective studies reporting post‐operative SAP including four RCTs and three observational studies were identified in the scoping review (Sørensen et al., 2024) (Table 7). Administration of post‐operative antimicrobial prophylaxis represented the intervention in all the included RCTs but none of the observational studies. The seven studies included a total of 1545 privately owned dogs and cats and there were no baseline differences regarding age, breed and sex between the intervention and comparator groups across the studies.

**Table 7:** Study characteristics for studies reporting incidence of surgical site infections in dogs and cats administered post‐operative antimicrobial prophylaxis versus dogs and cats not administered antimicrobial prophylaxis peri‐operatively (PICO 2)

Study	Subpopulation	Intervention			Comparator		Clinical outcome assessment	
		*n*	Substance	Dose, route	*n*	Placebo	Follow‐up	SSI definition
Aiken et al. (2015)*	S7 to S9	198	Cephalexin	20 mg/kg po bid 5 days	191	None	6 weeks	Purulent discharge/spontaneous dehiscence with serous drainage plus at least two inflammation signs /joint sepsis/a discharging sinus/infected implants diagnosed as a persistent lameness plus two of inflammation signs or radiographic signs of osteomyelitis simultaneously
Chutipongvivate et al. (2022)*	S1	244	Cephalexin	22.2 mg/kg po bid 7 days	248	None	30 days	Wound score and intervention
Pratesi et al. (2015)*	S7 to S9	46	Cephalexin or AMC	15 to 25 mg/kg or 12.5 mg/kg po bid 7 days	47	None	12 months	Deterioration after surgery for no other recognised reason and infection confirmed by microbial culture or considered present based on appropriate clinical signs and/or cytology
Spencer and Daye (2018)*	S9	70	Cefpodoxime	5 to 10 mg/kg po sid 7 days	64	po sid 7 days	8 weeks	Purulent discharge from incision, with or without laboratory confirmation, or which demonstrated three or more of the following if no drainage was present: redness, pain, swelling or heat at the incision Mild superficial dehiscence (<2 cm) without the presence of other clinical signs of infection was not considered to be SSI because these lesions healed on their own or with minimal medical intervention and no antibiotics
Andrade et al. (2016)	S8 and S9	33	Various	10 to 14 days	67	None	8 weeks	Positive microbial culture of joint fluid or wound exudate/purulent drainage/abscess/fistula/spontaneous dehiscence of one or more wound layers occurred with serous drainage. All incisions with drainage underwent bacteriological culture
Espinel‐Rupérez et al. (2019)	S2 to S4	126	NR	NR	58	No treatment	30 days	CDC criteria
Nazarali et al. (2015)	S9	79	NR	Median 10 days (12 hours to 21 days)	74	No treatment	8 weeks	CDC criteria

All studies administered peri‐operative antimicrobial prophylaxis

AM Antimicrobial; AMC Amoxicillin+clavulanic acid or potentiated amoxicillin; bid Twice daily; CDC Center for Disease Control and Prevention; iv Intravenous; NA Not applicable; NR Not reported; po Per oral; sc Subcutaneous; SSI Surgical site infection; S1 Subgroup 1 (neutering); S2 Subgroup 2 (other clean soft tissue); S3 Subgroup 3 (urologic); S4 Subgroup 4 (gastrointestinal); S5 Subgroup 5 (other clean‐contaminated soft tissue); S6 Subgroup 6 (contaminated); S7 Subgroup 7 (orthopaedic without implants); S8 Subgroup 8 (orthopaedic with implants); S9 Subgroup 9 (tibial plateau levelling osteotomy)

* Randomised controlled trials

Only two studies described soft tissue procedures (Chutipongvivate et al., 2022; Espinel‐Rupérez et al., 2019). Data could be extracted for specific subgroups (S3 and S4) after author contact (Espinel‐Rupérez et al., 2019). Five studies (3 RCT and 2 observational studies) represented dogs and cats undergoing orthopaedic procedures with implants (S8) or TPLO (S9). No studies were found where specific data could be extracted for dogs representing the S6 subgroup. Individual study characteristics are presented in Table 7. All patients in studies of post‐operative SAP were administered peri‐operative SAP.

#### Effect of post‐operative antimicrobial prophylaxis versus no antimicrobial prophylaxis

**FIG 5:**
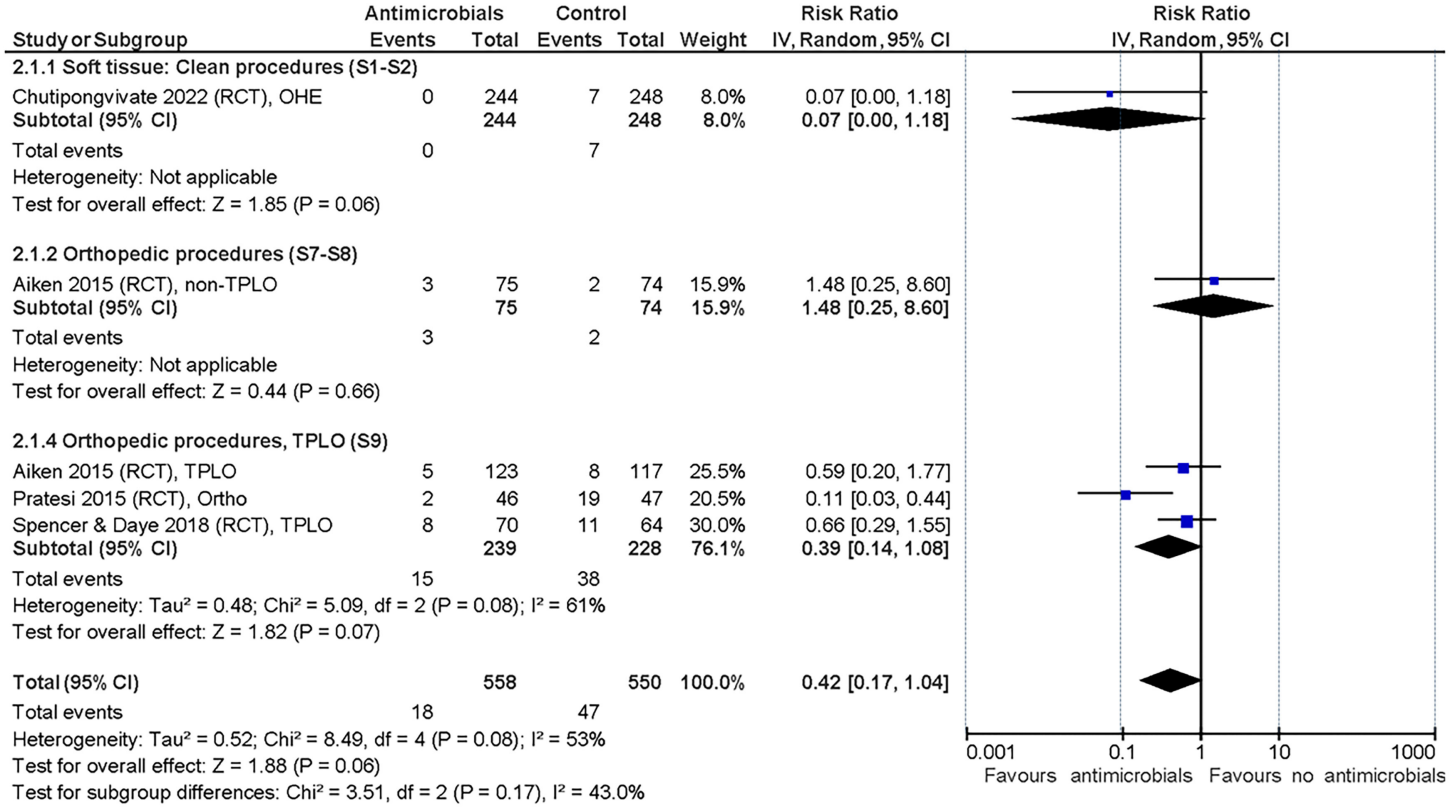
Randomised controlled trials included for evaluation of effect of post‐operative surgical antimicrobial prophylaxis (SAP) on surgical site infections (SSIs). Forest plot of the relative risk for SSI in dogs and cats administered post‐operative SAP versus dogs and cats not administered antimicrobial prophylaxis. Trials are subgrouped (S1 to S9) by surgery and wound classifications (Table 1). Individual trial results are shown as blue squares and pooled effects are shown as black diamonds. Relative risks are converted to absolute risks and related to clinical thresholds in Table 8. S1, Subgroup 1 (neutering); S2, Subgroup 2 (other clean soft tissue); S7, Subgroup 7 (orthopaedic without implants); S8, Subgroup 8 (orthopaedic with implants); and S9, Subgroup 9 (tibial plateau levelling osteotomy).

**FIG 6:**
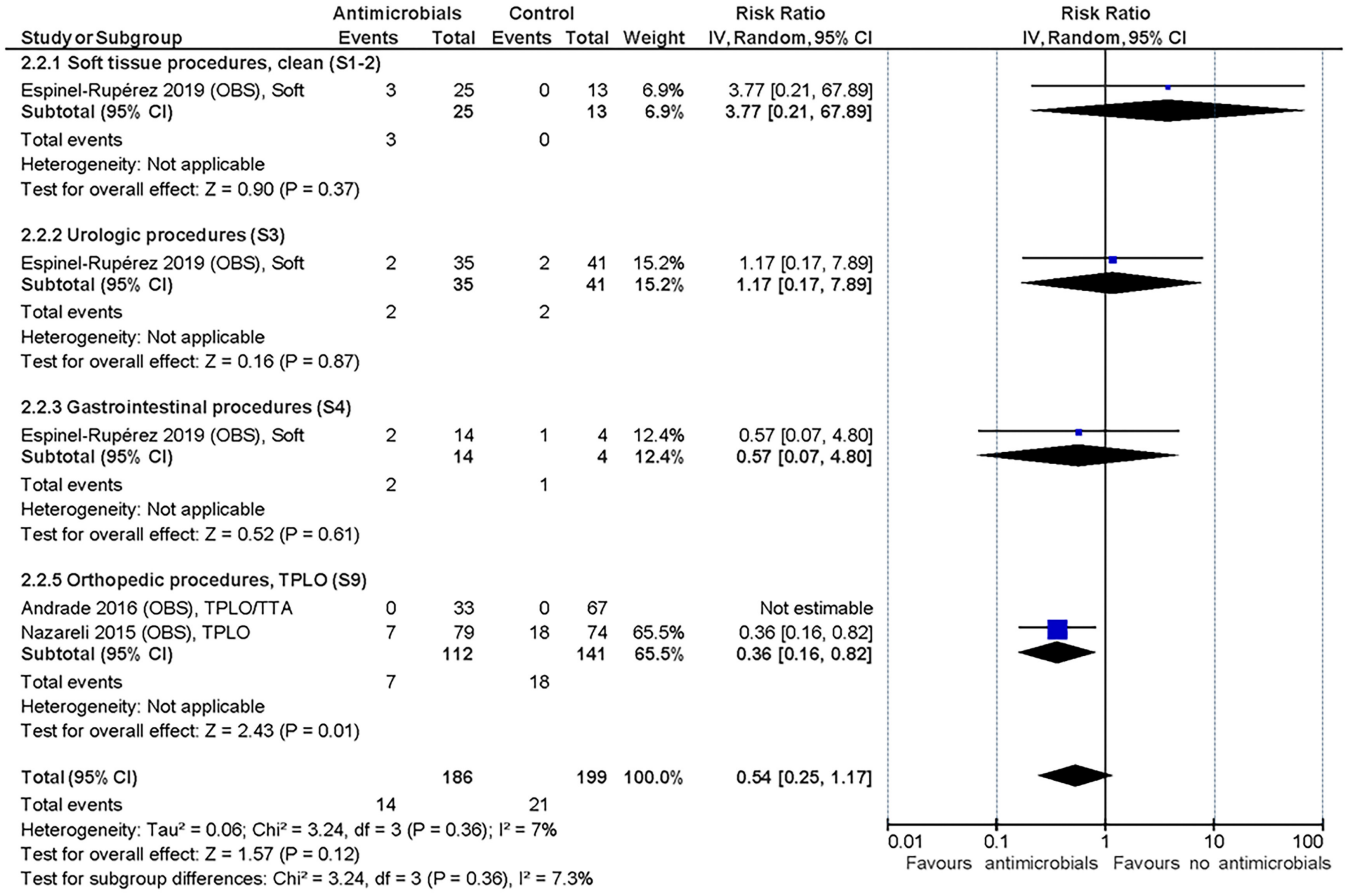
Observational studies included for evaluation of effect of post‐operative surgical antimicrobial prophylaxis (SAP) on surgical site infections (SSIs). Forest plot of the relative risk for SSI in dogs and cats administered post‐operative SAP versus dogs and cats not administered antimicrobial prophylaxis. Studies are subgrouped (S1 to S9) by surgery and wound classifications (Table 1). Individual study results are shown as blue squares and pooled effects are shown as black diamonds. Relative risks are converted to absolute risks and related to clinical thresholds in Table 9. S1, Subgroup 1 (neutering); S2, Subgroup 2 (other clean soft tissue); S3, Subgroup 3 (urologic); S4, Subgroup 4 (gastrointestinal); and S9, Subgroup 9 (tibial plateau levelling osteotomy).

The only RCT with clean, soft tissue procedures described OHE in cats (S1) and found a trivial effect of the post‐operative administration of SAP in favour of the intervention (Chutipongvivate et al., 2022). However, the few SSI events documented generates a very broad 95% CI for the risk ratio. The observational study not only represented a more diverse group of primarily clean soft tissue procedures but also found a trivial effect favouring no SAP administration (Espinel‐Rupérez et al., 2019). The three RCT and two observational studies describing exclusively TPLO procedures (S9) found a small positive effect overall, but with a wide range in the RCT data from trivial to moderate effect meaning that the certainty of evidence was downgraded for imprecision (Aiken et al., 2015; Andrade et al., 2016; Nazarali et al., 2015; Pratesi et al., 2015; Spencer & Daye, 2018). The summarised effects are shown in Figs 5 and 6 and absolute effects in Table 8 (SoF tables can be found in Supplementary file 2). Mortality was reported in all studies but was not linked to SSI development. No studies reported adverse effects attributable to antimicrobial use.

**Table 8:** The relative and absolute effects of post‐operative surgical antimicrobial prophylaxis versus no surgical antimicrobial prophylaxis

PICO 2	Study design	Risk ratio (95% CI)	*P*‐value	Absolute effect of post‐operative treatment	Clinical relevance*	Certainty of evidence
S1	RCT	0.07 [0.00 to 1.18]	.06	26 fewer SSI per 1000 animals (from 28 fewer to 5 more)	Trivial effect	Low
	OBS	3.77 [0.21 to 67.89]	.37	0 fewer SSI per 1000 animals (from 0 fewer to 4 more)	Trivial effect	Very low
S2	RCT	0.07 [0.00 to 1.18]	.06	26 fewer SSI per 1000 animals (from 28 fewer to 5 more)	Trivial effect	Very low
	OBS	3.77 [0.21 to 67.89]	.37	0 fewer SSI per 1000 animals (from 0 fewer to 4 more)	Trivial effect	Very low
S3	RCT	0.07 [0.00 to 1.18]	.06	26 fewer SSI per 1000 animals (from 28 fewer to 5 more)	Trivial effect	Very low
	OBS	1.17 [0.17 to 7.89]	.87	8 more SSI per 1000 animals (from 40 fewer to 336 more)	Trivial effect	Very low
S4	RCT	0.07 [0.00 to 1.18]	.06	26 fewer SSI per 1000 animals (from 28 fewer to 5 more)	Trivial effect	Very low
	OBS	0.57 [0.07 to 4.80]	.61	108 fewer SSI per 1000 animals (from 232 fewer to 950 more)	Moderate effect	Very low
S5	RCT	0.07 [0.00 to 1.18]	.06	26 fewer SSI per 1000 animals (from 28 fewer to 5 more)	Trivial effect	Very low
	OBS	1.99 [0.59 to 6.73]	NA	51 fewer SSI per 1000 animals (from 21 fewer to 296 more)	Small effect	Very low
S6	RCT	0.07 [0.00 to 1.18]	.06	26 fewer SSI per 1000 animals (from 28 fewer to 5 more)	NA	Very low
	OBS	1.99 [0.59 to 6.73]	NA	51 fewer SSI per 1000 animals (from 21 fewer to 296 more)	NA	Very low
S7	RCT	1.48 [0.25 to 8.60]	.66	13 more SSI per 1000 animals (from 20 fewer to 205 more)	Trivial effect	Low
S8	RCT	1.48 [0.25 to 8.60]	.66	13 more SSI per 1000 animals (from 20 fewer to 205 more)	Trivial effect	Moderate
S9	RCT	0.39 [0.14 to 1.08]	.07	102 fewer SSI per 1000 animals (from 143 fewer to 13 more)	Small effect	Very low
	OBS	0.36 [0.16 to 0.82]	.01	82 fewer SSI per 1000 animals (from 107 fewer to 23 more)	Small effect	Very low

The clinical relevance is interpreted according to predetermined thresholds (Table 3) and the certainty of evidence according to the GRADE approach (Table 4)

CI Confidence interval; GRADE Grading of Recommendations Assessment, Development and Evaluation; NA Not applicable; OBS Observational trial; RCT Randomised controlled trial; S1, Subgroup 1 (neutering); S2, Subgroup 2 (other clean soft tissue); S3, Subgroup 3 (urologic); S4, Subgroup 4 (gastrointestinal); S5, Subgroup 5 (other clean‐contaminated soft tissue); S7, Subgroup 7 (orthopaedic without implants); S8, Subgroup 8 (orthopaedic with implants); and S9, Subgroup 9 (tibial plateau levelling osteotomy); SSI Surgical site infection.

* The clinical relevance of the estimated absolute effect is interpreted according to the thresholds selected by the veterinarians/stakeholders

#### Certainty of evidence

**FIG 7:**
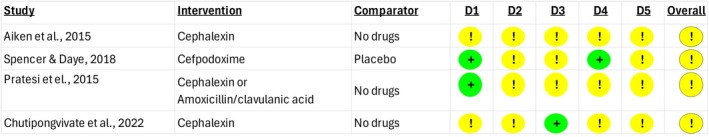
Risk of bias analysis for randomised controlled trials included in the review on post‐operative administration of antimicrobials for surgical site infection prophylaxis. D1, Randomisation process; D2, Deviations from the intended interventions; D3, Missing outcome data; D4, Measurement of the outcome; and D5, Selection of the reported result. Green dots indicate low risk of bias, yellow dots some concerns and red dots high risk of bias.

For the four RCT studies describing post‐operative SAP, there were concerns of bias, primarily because of lack of reporting relevant details on methodology as shown in Fig 7.

Therefore, the risk of bias was downgraded by one level in the certainty assessment. All RCT were deemed to have serious risk of bias and all observational studies were defined as very serious risk of bias. The overall certainty of evidence was very low in all subgroups except for S1, S7 and S8. This was primarily due to the serious, or very serious, risk of bias but also serious, or extremely serious, imprecision assessment. Not all surgical subgroups were represented or could be extracted from the published data (Table 7, 8).

## DISCUSSION

The aim of this systematic review was to provide some first objective measures to help clinicians determine the need for peri‐ and post‐operative SAP.

The aggregated data from the RCT and observational studies suggest a marginal benefit (reduction in SSI risk) from peri‐operative SAP. However, in both instances, the summary statistic crosses the line of null effect indicating that the result is not statistically significant. More importantly, only a few studies crossed the line of clinically relevant effect according to the predetermined clinical thresholds. Similarly, for post‐operative SAP, no clinical benefit was demonstrated in either the RCT or the observational studies. The trivial effect of SAP shown by most of the included studies represents a weak argument to administer SAP in most cases. Cost–benefit analysis weighs harm and adverse effects against this trivial benefit. Unfortunately, adverse effects attributable to SAP were not well reported, making it difficult to qualify individual patient costs of treatment.

All included studies were considered to have serious or very serious risks of bias leading to a downgrading of the certainty of evidence. Many of the poor scores assigned were due to a lack of reporting of methodological details. The publication of some articles more than 20 years ago may have contributed to the absence of such information. Publication standards evolve with time; while older studies may have been well conducted, the absence of specific details routinely expected today contributed to the low ROB scores.

Few studies were eligible for inclusion in this systematic review (Sørensen et al., 2024). And efforts were made to subgroup the dataset into meaningful sections where findings could be expected to apply similarly. Since many common procedures were not represented in the available dataset, broad categories were created based on surgical wound class. However, this approach introduces serious indirectness imposing frequent downgrading of the certainty of evidence. While studies in people have shown reasonable correlation between wound classification and SSI rate (Culver et al., 1991; Ortega et al., 2012) with established estimates of SSI rates for clean (1% to 5%), clean‐contaminated (3% to 11%), contaminated (10% to 17%) and dirty (>27%) procedures (Townsend et al., 2007), some procedure‐specific risk factors may be overlooked or exaggerated by including distinctly different procedures in the same wound classification. Furthermore, considerable intraclass variability should be anticipated as the degree of wound contamination (microbial burden) varies considerably according to surgical site (Mioton et al., 2013; Onyekwelu et al., 2017; Wilke et al., 2022). These considerations underscore the need for additional studies addressing the utility of SAP for those procedures that are most frequently performed in veterinary practice.

The degree of stakeholder engagement during the development of 15 European antimicrobial guidelines was highly variable (Allerton et al., 2021). This step is nonetheless crucial to maximise guideline implementation (Petkovic et al., 2020). Recommendations and outcomes should be relevant to key stakeholders, to influence their antimicrobial prescribing habits (Wathne et al., 2018). Furthermore, greater awareness of antimicrobial use guidelines has been shown to correlate with improved antimicrobial stewardship tendency (Farrell et al., 2024). It is therefore vital that guidelines are developed with the end user in mind. In this systematic review, the incorporation of thresholds derived from interviews with veterinary practitioners (the target user of SAP guidelines) offers a measure of the acceptability of the accumulative result to the target audience. Precision is deemed adequate if the conclusion would not differ if the upper or lower boundary of the confidence interval represented the true effect (Schünemann et al., 2013).

The stakeholder group consisted of volunteers with surgical experience who expressed an interest in completing the threshold survey. This could introduce a selection bias distorting the responses and producing a non‐representative sample (Higgins et al., 2011) as those with an interest in AMR and antimicrobial stewardship may be more likely to contribute. Nonetheless, considerable heterogeneity was observed in the responses reflecting a range of viewpoints and willingness to accept different SSI rates. Ethnographic studies have shown stark differences in attitude to antimicrobial use between acute surgical and acute medical teams (Charani, Ahmad, et al., 2019). In human hospitals, surgeons have been found to prescribe antimicrobials more frequently and for longer courses than their medical colleagues (Charani, de Barra, et al., 2019). Given the wide range of roles undertaken in primary veterinary care combining both medical and surgical responsibilities, a range of opinions and perspectives on the weighing of antimicrobial benefits and harms may be expected. Running the survey with a larger sample size or incorporating antimicrobial harms effect could have narrowed or broadened the thresholds, or allow stratification according to respondent characteristics (e.g. level of surgical experience, orthopaedic or soft tissue preference).

Recent European legislation aimed at reducing antimicrobial use, stipulates that antibiotic medicinal products must not be used prophylactically except in exceptional circumstances, and only for individual animals, where the risk of infection or infectious disease is very high and the consequences of not prescribing are likely to be severe (EU, 2022; Veterinary Medicines (Amendment etc.) Regulations, 2024). As SAP remains legally permissible under these conditions, the critical question becomes how to define an exceptional case. To support rational and evidence‐based decision‐making, veterinarians require robust data demonstrating the benefits and harms of SAP for specific surgical procedures in different clinical contexts. The findings of the present systematic review should therefore be applied within this regulatory framework, providing an evidence base for clinical protocols that align with current legal and ethical standards for antimicrobial stewardship.

The small number of suitable studies reflects a major knowledge gap and more research is required to bolster the evidence base. A reliance on RCTs as the reference standard may introduce barriers to the collection of this data. RCTs are time consuming and expensive to run with only one placebo‐controlled RCT identified from the last 20 years (Spencer & Daye, 2018). Greater incorporation of observational data (both prospective and retrospective) may offer a more achievable means of answering some of these questions. As a concluding remark, rational clinical decision‐making is based upon the balance between benefits and harms. Regardless of study design, future studies are strongly encouraged to measure and report any observed adverse effects of SAP treatment along with the beneficial effects.

In conclusion, very low to moderate certainty evidence showed that SAP only had a trivial or small clinical effect on SSI incidence in all surgical procedures. No adverse effects were reported in any of the studies.

## Author contributions

All authors (T. M. S., K. S., J. S. W., F. A. and L. R. J.) contributed to conception or design of the work, selection of PICOs and outcomes, data collection, critical revision of the article and final approval of the version to be published. T. M. S. conducted the meta‐analysis and drafted the manuscript. K. S. and L. R. J assessed the risk of bias and contributed to the manuscript. F. A. conducted the threshold survey and co‐drafted the manuscript. J. S. W. contributed to the manuscript.

## Conflict of interest

This article is based upon work from the COST Action European Network for Optimization of Veterinary Antimicrobial Treatment (CA18217), supported by COST (European Cooperation in Science and Technology). None of the authors of this paper has a financial or personal relationship with other people or organisations that could inappropriately influence or bias the content of the paper.

## Supporting information


**Supplementary file 1.** Threshold interview guide.

**Supplementary file 2.** Summary of findings (SoF) tables.

**Supplementary file 3.** Certainty of evidence decisions (CoE).

## Data Availability

The data that support the findings of this study are available from the corresponding author upon reasonable request.
