# Supplementary material for: Efficacy of antimicrobial prophylaxis on the risk of surgical site infections in companion animal surgery: a systematic review and meta‐analysis for European Network for Optimization of Antimicrobial Therapy (ENOVAT) guidelines
Source: J Small Anim Pract. 2026 Feb 18;67(3):198–211. doi: 10.1111/jsap.70055 (PMC12968480; doi:10.1111/jsap.70055)
Supplement: Supplementary file 1 — Threshold interview guide. [file JSAP-67-198-s001.docx]

Content and purpose of this survey: We will present you with the key benefits (absolute risk reduction of SSI) and harms of antimicrobial use in cats and dogs undergoing surgery. We will then ask you for your perspective on the use of perioperative/postoperative antimicrobials under each scenario. Each question will vary the risk reduction of SSI. Please read these carefully.

We will use your responses to inform our discussion of the tipping point, with regard to baseline SSI (superficial, deep, organ space), at which the majority would switch from declining to accepting antimicrobials.

1. For cats and dogs undergoing a soft tissue surgery, the baseline risk of **superficial** SSI is estimated at 5 %, meaning that 50 of 1000 animals develop a superficial SSI.

By superficial SSI we mean involvement of the **superficial parts of the wound** (skin/mucous membranes and subcutaneous tissue of the incision).

In a hypothetical scenario the prophylactic treatment of 1000 animals with **perioperative** antimicrobials reduces the risk of SSIs from 50 to 0 animals. The harms of antimicrobials include gastrointestinal upset and other side effects, dysbiosis, antimicrobial resistance (both for the individual cat/dog and public health).

How do you view the tradeoff between benefits and harms? Does this SSI risk-reduction justify the use of prophylactic perioperative antimicrobials? (Yes/No)

If not: what risk reduction would be the tipping point where you would consider the use of prophylactic antimicrobials (what number in a thousand animals)?

If yes: what risk reduction would be the tipping point where you would consider withholding prophylactic antimicrobials (what number in a thousand animals)? In other words when would the risk reduction be too trivial and the harm too big for you to use **perioperative** antimicrobials?

1. Same questions for **postoperative** antimicrobials with a risk reduction of SSIs from 50 to 5 animals.
2. Staying with the superficial SSIs we will now ask you the same questions but for orthopaedic surgery.

We are now going to investigate your perspective on antimicrobial use with regard to deep SSIs. By deep SSI we mean involvement of the **deep tissues of the incision** (e.g. fascia and/or muscle).

1. For cats and dogs undergoing abdominal surgery, such as a spey (OVH or an exploratory laparotomy) the baseline risk of **deep** SSI is estimated at 3 %, meaning that 30 of 1000 animals develop a deep SSI.

In a hypothetical scenario the prophylactic treatment of 1000 animals with **perioperative** antimicrobials reduces the risk of **deep SSIs** from 30 to 10 animals.

1. Same questions for **postoperative** antimicrobials with a risk reduction of SSIs from 30 to 10 animals.

We are now going to investigate your perspective on antimicrobial use with regard to **organ/space** SSIs. By organ/space SSI we mean involvement of any part of the body deeper than the fascial/muscle layers, which was opened or manipulated during the operative procedure, including bone and organs.

For cats and dogs undergoing urological surgery, such as cystotomy, the baseline risk of **organ/space** SSI is estimated at 5 %, meaning that 50 of 1000 animals develop a organ/space SSI.

In a hypothetical scenario the prophylactic treatment of 1000 animals with **perioperative** antimicrobials reduces the risk of **organ/space SSIs** from 50 to 10 animals.

1. Same questions for **postoperative** antimicrobials with a risk reduction of SSIs from 50 to 10 animals.
2. Staying with the **organ/space** SSIs we will now ask you the same questions for other types of surgery.

We are now going to investigate your perspective on antimicrobial use with regard to **implant-associated** SSIs. By **implant-associated** SSI we mean any of the other SSI classifications that have shown to include / spread towards the implant.

1. For cats and dogs undergoing soft tissue surgery, such as a TPLO, the baseline risk of **implant-associated** SSI is estimated at 5 %, meaning that 50 of 1000 animals develop a deep SSI.

In a hypothetical scenario the prophylactic treatment of 1000 animals with **perioperative** antimicrobials reduces the risk of **implant-associated SSIs** from 50 to 10 animals.

1. Same questions for **postoperative** antimicrobials with a risk reduction of SSIs from 50 to 0 animals.

| **Type of surgery** | **Peri or post** | **SSI type (superficial, deep, organ/space or implant- associated)** | **Baseline level (source)** | **Risk reduction**  **(in absolute numbers if 1000 animals are treated with AMs)** | **Does this SSI risk-reduction justify the antimicrobials?**  **Yes or No** | **If NO: what risk reduction would be the tipping point where you would consider the use of antimicrobials (what number in a thousand animals)?** | **If YES: what risk reduction would be the tipping point where you would consider withholding prophylactic antimicrobials (what number in a thousand animals)? In other words when would the risk reduction be too trivial and the harm too big for you to use SAP?** |
| --- | --- | --- | --- | --- | --- | --- | --- |
| Soft tissue surgery | Peri | Superficial | 5% | 50 to 5 |  |  |  |
| Soft tissue surgery | Post | Superficial | 5% | 50 to 5 |  |  |  |
| Orthopaedic surgery | Peri | Superficial | 5% | 50 to 5 |  |  |  |
| Orthopaedic surgery | Post | Superficial | 5% | 50 to 5 |  |  |  |
| Abdominal surgery (e.g., OVH, enterotomy or cystotomy) | Peri | Deep | 3% | 30 to 5 |  |  |  |
| Abdominal surgery (e.g., OVH, enterotomy or cystotomy) | Post | Deep | 3% | 30 to 5 |  |  |  |
| Elective surgery (e.g., OVH) e.g., stump pyometra | Peri | Organ/Space | 1% | 10 to 5 |  |  |  |
| Elective surgery (e.g., OVH) e.g., stump pyometra | Post | Organ/Space | 1% | 10 to 5 |  |  |  |
| GI surgery (e.g., foreign body removal) → septic peritonitis | Peri | Organ/Space | 3-12% (Swinbourne et al. 2017, Shales et al. 2010) | 50 to 10 |  |  |  |
| GI surgery (e.g., foreign body removal) → septic peritonitis | Post | Organ/Space | 3-12% (Swinbourne et al. 2017, Shales et al. 2010) | 50 to 10 |  |  |  |
| Urological sx (e.g., cystotomy) | Peri | Organ/Space | 5% (Appel et al. 2012) | 50 to 10 |  |  |  |
| Urological sx (e.g., cystotomy) | Post | Organ/Space | 5% (Appel et al. 2012) | 50 to 10 |  |  |  |
| Ortho sx (without implants) e.g., septic arthritis post arthrotomy | Peri | Organ/Space | 4% (Billings et al, 1990)  Likely <1% (personal communication) | 40 to 10 |  |  |  |
| Ortho sx (without implants) e.g., septic arthritis post arthrotomy | Post | Organ/Space | 4% (Billings et al, 1990)  Likely <1% (personal communication) | 40 to 10 |  |  |  |
| Ortho sx (with implants) e.g., TPLO | Peri | Implant-associated | 3-8% (McDouggal et al. 2021, Husi et al. 2023) | 50 to 10 |  |  |  |
| Ortho sx (with implants) e.g., TPLO | Post | Implant-associated | 3-8% (McDouggal et al. 2021, Husi et al. 2023) | 50 to 10 |  |  |  |

**References:**

1. Swinbourne F, Jeffery N, Tivers MS, Artingstall R, Bird F, Charlesworth T, et al. The incidence of surgical site dehiscence following full-thickness gastrointestinal biopsy in dogs and cats and associated risk factors. J Small Anim Pract. 2017 Sep;58(9):495–503.

2. Appel S, Otto SJ, Weese JS. Cystotomy practices and complications among general small animal practitioners in Ontario, Canada. Can Vet J. 2012 Mar;53(3):303–10.

3. Billings L, Vasseur PB, Fancher C, Miller M, Nearenberg D. Wound infection rates in dogs and cats after use of cotton muslin or disposable impermeable fabric as barrier material: 720 cases (1983-1989). J Am Vet Med Assoc. 1990 Oct 1;197(7):889–92.

4. McDougall RA, Spector DI, Hart RC, Dycus DL, Erb HN. Timing of and risk factors for deep surgical site infection requiring implant removal following canine tibial plateau leveling osteotomy. Vet Surg. 2021 Jul;50(5):999–1008.

5. Husi B, Overesch G, Forterre F, Rytz U. Surgical site infection after 769 Tibial Plateau Leveling Osteotomies. Front Vet Sci. 2023;10:1133813.
