# Supplementary material for: Efficacy of antimicrobial prophylaxis on the risk of surgical site infections in companion animal surgery: a systematic review and meta‐analysis for European Network for Optimization of Antimicrobial Therapy (ENOVAT) guidelines
Source: J Small Anim Pract. 2026 Feb 18;67(3):198–211. doi: 10.1111/jsap.70055 (PMC12968480; doi:10.1111/jsap.70055)
Supplement: Supplementary file 2 — Summary of findings (SoF) tables. [file JSAP-67-198-s003.docx]

SoF Table 1: Summary of findings for PICO 1 relating to peri-operative surgical Antimicrobial Prophylaxis (SAP) compared to no SAP in dogs and cats.

| **Peri-op AM compared to no AM for SAP in dogs and cats** | | | | | |
| --- | --- | --- | --- | --- | --- |
| **Outcomes** | **№ of participants (studies) Follow-up** | **Certainty of the evidence (GRADE)** | **Relative effect (95% CI)** | **Anticipated absolute effects** | |
|  |  |  |  | **Risk with no AM** | **Risk difference with Peri-op AM** |
| SSI, S1 | 835 (1 RCT) | ⨁⨁◯◯ Low | **RR 0.88** (0.57 to 1.37) | 93 per 1,000 | **11 fewer per 1,000** (40 fewer to 34 more) |
| SSI, S1 | 1343 (3 non-randomised studies) | ⨁◯◯◯ Very low | **RR 0.58** (0.23 to 1.45) | 40 per 1,000 | **17 fewer per 1,000** (31 fewer to 18 more) |
| SSI, S2 | 835 (1 RCT) | ⨁◯◯◯ Very low | **RR 0.88** (0.57 to 1.37) | 93 per 1,000 | **11 fewer per 1,000** (40 fewer to 34 more) |
| SSI, S2 | 1343 (3 non-randomised studies) | ⨁◯◯◯ Very low | **RR 0.58** (0.23 to 1.45) | 40 per 1,000 | **17 fewer per 1,000** (31 fewer to 18 more) |
| SSI, S3-S5 | 38 (1 RCT) | ⨁◯◯◯ Very low | **RR 1.35** (0.25 to 7.19) | 111 per 1,000 | **39 more per 1,000** (83 fewer to 688 more) |
| SSI, S3 | 964 (2 non-randomised studies) | ⨁◯◯◯ Very low | **RR 0.66** (0.24 to 1.85) | 77 per 1,000 | **26 fewer per 1,000** (58 fewer to 65 more) |
| SSI, S4-S5 | 964 (2 non-randomised studies) | ⨁◯◯◯ Very low | **RR 0.66** (0.24 to 1.85) | 77 per 1,000 | **26 fewer per 1,000** (58 fewer to 65 more) |
| SSI, S6 | 16 (1 non-randomised study) | ⨁◯◯◯ Very low | **RR 0.56** (0.04 to 7.83) | 286 per 1,000 | **126 fewer per 1,000** (274 fewer to 1,951 more) |
| SSI, S7-S9 | 314 (3 RCTs) | ⨁◯◯◯ Very low | **RR 0.38** (0.13 to 1.13) | 54 per 1,000 | **34 fewer per 1,000** (47 fewer to 7 more) |
| SSI, S7-S8 | 1776 (2 non-randomised studies) | ⨁◯◯◯ Very low | **RR 0.62** (0.29 to 1.33) | 50 per 1,000 | **19 fewer per 1,000** (35 fewer to 16 more) |
| SSI, S9 | 1776 (2 non-randomised studies) | ⨁◯◯◯ Very low | **RR 0.62** (0.29 to 1.33) | 50 per 1,000 | **19 fewer per 1,000** (35 fewer to 16 more) |
| ***The risk in the intervention group** (and its 95% confidence interval) is based on the assumed risk in the comparison group and the **relative effect** of the intervention (and its 95% CI).  **CI:** confidence interval; **RR:** risk ratio; SSI: surgical site infection; RCT; randomized control trial; AM: antimicrobial | | | | | |
| **GRADE Working Group grades of evidence** **High certainty:** confidence that the true effect lies close to that of the estimate of the effect. **Moderate certainty:** moderately confidence in the effect estimate: the true effect is likely to be close to the estimate of the effect, but there is a possibility that it is substantially different. **Low certainty:** the confidence in the effect estimate is limited: the true effect may be substantially different from the estimate of the effect. **Very low certainty:** very little confidence in the effect estimate: the true effect is likely to be substantially different from the estimate of effect. | | | | | |

SoF Table 2: Summary of findings for PICO 2 relating to post-operative SAP compared to no SAP in dogs and cats.

| **Post-op AM compared to no AM for SAP in dogs and cats?** | | | | | |
| --- | --- | --- | --- | --- | --- |
| **Outcomes** | **№ of participants (studies) Follow-up** | **Certainty of the evidence (GRADE)** | **Relative effect (95% CI)** | **Anticipated absolute effects** | |
|  |  |  |  | **Risk with no AM** | **Risk difference with Post-op AM** |
| SSI, S1 | 492 (1 RCT) | ⨁◯◯◯ Very low | **RR 0.07** (0.00 to 1.18) | 28 per 1,000 | **26 fewer per 1,000** (28 fewer to 5 more) |
| SSI, S1 | 97 (1 non-randomised study) | ⨁◯◯◯ Very low | **RR 3.94** (0.24 to 64.48) | 0 per 1,000 | **0 fewer per 1,000** (0 fewer to 4 fewer) |
| SSI, S2 | 492 (1 RCT) | ⨁◯◯◯ Very low | **RR 0.07** (0.00 to 1.18) | 28 per 1,000 | **26 fewer per 1,000** (28 fewer to 5 more) |
| SSI, S2 | 97 (1 non-randomised study) | ⨁◯◯◯ Very low | **RR 3.94** (0.24 to 64.48) | 0 per 1,000 | **0 fewer per 1,000** (0 fewer to 4 fewer) |
| SSI, S3-S6 | 492 (1 RCT) | ⨁◯◯◯ Very low | **RR 0.07** (0.00 to 1.18) | 28 per 1,000 | **26 fewer per 1,000** (28 fewer to 5 more) |
| SSI, S3 | 76 (1 non-randomised study) | ⨁◯◯◯ Very low | **RR 1.17** (0.17 to 7.89) | 49 per 1,000 | **8 more per 1,000** (40 fewer to 336 more) |
| SSI, S4 | 18 (1 non-randomised study) | ⨁◯◯◯ Very low | **RR 0.57** (0.07 to 4.80) | 250 per 1,000 | **108 fewer per 1,000** (232 fewer to 950 more) |
| SSI, S5-S6 | 184 (1 non-randomised study) | ⨁◯◯◯ Very low | **RR 1.99** (0.59 to 6.73) | 52 per 1,000 | **51 more per 1,000** (21 fewer to 296 more) |
| SSI, S7 | 149 (1 RCT) | ⨁⨁◯◯ Low | **RR 1.48** (0.25 to 8.60) | 27 per 1,000 | **13 more per 1,000** (20 fewer to 205 more) |
| SSI, S8 | 149 (1 RCT) | ⨁⨁⨁◯ Moderate | **RR 1.48** (0.25 to 8.60) | 27 per 1,000 | **13 more per 1,000** (20 fewer to 205 more) |
| SSI, S9 | 467 (3 RCTs) | ⨁◯◯◯ Very low | **RR 0.39** (0.14 to 1.08) | 167 per 1,000 | **102 fewer per 1,000** (143 fewer to 13 more) |
| SSI, S9 | 253 (2 non-randomised studies) | ⨁◯◯◯ Very low | **RR 0.36** (0.16 to 0.82) | 128 per 1,000 | **82 fewer per 1,000** (107 fewer to 23 fewer) |
| ***The risk in the intervention group** (and its 95% confidence interval) is based on the assumed risk in the comparison group and the **relative effect** of the intervention (and its 95% CI).  **CI:** confidence interval; **RR:** risk ratio; SSI: surgical site infection; RCT; randomized control trial; AM: antimicrobial | | | | | |
| **GRADE Working Group grades of evidence** **High certainty:** confidence that the true effect lies close to that of the estimate of the effect. **Moderate certainty:** moderately confidence in the effect estimate: the true effect is likely to be close to the estimate of the effect, but there is a possibility that it is substantially different. **Low certainty:** the confidence in the effect estimate is limited: the true effect may be substantially different from the estimate of the effect. **Very low certainty:** very little confidence in the effect estimate: the true effect is likely to be substantially different from the estimate of effect. | | | | | |
