# Supplementary material for: Efficacy of antimicrobial prophylaxis on the risk of surgical site infections in companion animal surgery: a systematic review and meta‐analysis for European Network for Optimization of Antimicrobial Therapy (ENOVAT) guidelines
Source: J Small Anim Pract. 2026 Feb 18;67(3):198–211. doi: 10.1111/jsap.70055 (PMC12968480; doi:10.1111/jsap.70055)
Supplement: Supplementary file 3 — Certainty of evidence decisions (CoE). [file JSAP-67-198-s002.docx]

**CoE Table 1**. Certainty of evidence for PICO 1 subgroups. RCT and observational studies separated.

| Subgroup | Study design | Risk of bias | Inconsistency | Indirectness | Imprecision | Other | Overall certainty |
| --- | --- | --- | --- | --- | --- | --- | --- |
| S1 | RCT | Very serious | Not serious | Not serious | Not serious | None | Low |
|  | OBS | Very serious | Not serious | Not serious | Not serious | None | Very low |
| S2 | RCT | Very serious | Not serious | Serious^a^ | Not serious | None | Very low |
|  | OBS | Very serious | Not serious | Not serious | Serious^f^ | None | Very low |
| S3 | RCT | Very serious | Not serious | Not serious | Extremely serious^h^ | None | Very low |
|  | OBS | Very serious | Not serious | Serious^b^ | Extremely serious^h^ | None | Very low |
| S4 | RCT | Very serious | Not serious | Not serious | Extremely serious^h^ | None | Very low |
|  | OBS | Very serious | Not serious | Serious^c^ | Extremely serious^g^ | None | Very low |
| S5 | RCT | Very serious | Not serious | Not serious | Extremely serious^h^ | None | Very low |
|  | OBS | Very serious | Not serious | Serious^d^ | Extremely serious^g^ | None | Very low |
| S6 | OBS | Very serious | Not serious | Not serious | Extremely serious^h^ | None | Very low |
| S7 | RCT | Very serious | Not serious | Not serious | Serious^f^ | None | Very low |
|  | OBS | Very serious | Not serious | Not serious | Serious^f^ | None | Very low |
| S8 | RCT | Very serious | Not serious | Not serious | Serious^f^ | None | Very low |
|  | OBS | Very serious | Not serious | Serious^e^ | Serious^f^ | None | Very low |
| S9 | RCT | Very serious | Not serious | Not serious | Serious^f^ | None | Very low |
|  | OBS | Very serious | Not serious | Not serious | Serious^f^ | None | Very low |

OBS, Observational studies; S1, Neutering; S2, Other clean soft tissue procedures; S3, Urologic; S4, Gastrointestinal; S5, Other clean-contaminated procedures; S6: Contaminated soft tissue procedures; S7: Orthopedic procedures without implants; S8, Orthopedic procedures with implants; S9, Tibial Plateau Leveling Osteotomy; RCT, Randomized Controlled Trials.

^a^ The majority (524/621) of procedures in single relevant RCT related to procedures for P1 hence indirectness was downgraded one level.

^b^ Urinary procedures were not specifically described so it is impossible to determine how many of the procedures related to this organ system in any of the evaluated observational studies, indirectness was downgraded one level.

^c^ Gastrointestinal procedures were not specifically described so it is impossible to determine how many of the procedures related to this organ system in any of the evaluated observational studies, indirectness was downgraded one level.

^d^ Other (non-urinary or non-gastrointestinal) procedures were not specifically described so it is impossible to determine how many of the procedures related to this organ system in any of the evaluated observational studies, indirectness was downgraded one level.

^e^ The only observational data was a mixed group of procedures both regarding soft tissue and orthopaedic and wound classifications hence indirectness was downgraded one level.

^f^ As confidence intervals crosses one threshold level, imprecision was downgraded one level.

^g^ As confidence intervals crosses two threshold level, imprecision was downgraded two levels.

^h^ As confidence intervals crosses three threshold level, imprecision was downgraded three levels.

**CoE Table 2**. Certainty of evidence for PICO 2 subgroups. RCT and observational studies separated.

| Subgroup | Study design | Risk of bias | Inconsistency | Indirectness | Imprecision | Other considerations | Overall certainty |
| --- | --- | --- | --- | --- | --- | --- | --- |
| S1 | RCT | Serious | Not serious | Serious | Not serious | None | Low |
|  | OBS | Very serious | Not serious | Serious | Not serious | None | Very low |
| S2 | RCT | Serious | Not serious | Serious | Serious^a^ | None | Very low |
|  | OBS | Very serious | Not serious | Not serious | Not serious | None | Very low |
| S3 | RCT | Serious | Not serious | Serious | Extremely serious^c^ | None | Very low |
|  | OBS | Very serious | Not serious | Not serious | Not serious | None | Very low |
| S4 | RCT | Serious | Not serious | Serious | Extremely serious^c^ | None | Very low |
|  | OBS | Very serious | Not serious | Not serious | Not serious | None | Very low |
| S5 | RCT | Serious | Not serious | Serious | Extremely serious^c^ | None | Very low |
|  | OBS | Very serious | Not serious | Not serious | Not serious | None | Very low |
| S6 | RCT | Serious | Not serious | Serious | Extremely serious^c^ | None | Very low |
|  | OBS | Very serious | Not serious | Not serious | Not serious | None | Very low |
| S7 | RCT | Serious | Not serious | Serious | Not serious | None | Low |
| S8 | RCT | Serious | Not serious | Not serious | Not serious | None | Moderate |
| S9 | RCT | Serious | Not serious | Not serious | Very serious^b^ | None | Very low |
|  | OBS | Very serious | Not serious | Not serious | Not serious | None | Very low |

OBS, Observational studies; S1, Neutering; S2, Other clean soft tissue procedures; S3, Urologic; S4, Gastrointestinal; S5, Other clean-contaminated procedures; S6: Contaminated soft tissue procedures; S7: Orthopedic procedures without implants; S8, Orthopedic procedures with implants; S9, Tibial Plateau Leveling Osteotomy; OBS, Observational trial; RCT, Randomized Controlled Trials.

^a^ As confidence intervals crosses one threshold level, imprecision was downgraded one level.

^b^ As confidence intervals crosses two threshold level, imprecision was downgraded two levels.

^c^ As confidence intervals crosses three threshold level, imprecision was downgraded three levels.
